# Immunomodulation in Pediatric Sepsis: A Narrative Review

**DOI:** 10.3390/jcm14092983

**Published:** 2025-04-25

**Authors:** Gabriella Bottari, Fabio Silvio Taccone, Angelica Corrias, Mariangela Irrera, Paolo Currao, Michele Salvagno, Corrado Cecchetti, Didier Payen

**Affiliations:** 1 Pediatric Intensive Care Unit, Children Hospital Bambino Gesù, IRCSS, 00165 Rome, Italy; corrado.cecchetti@opbg.net; 2 Department of Intensive Care, Hopital Universitaire de Bruxelles (HUB), Université Libre de Bruxelles (ULB), 1050 Brussels, Belgium; fabio.taccone@hubruxelles.be (F.S.T.); michele.salvagno@ulb.be (M.S.); 3 Pediatric Clinic, “Microcitemico—A. Cao” Pediatric Hospital, University of Cagliari, 09124 Cagliari, Italy; angelicacorrias03@gmail.com (A.C.); paolocurrao@gmail.com (P.C.); 4 Academy of Pediatrics, Bambino Gesù Children’s Hospital, IRCCS, 00165 Rome, Italy; mariangela.irrera@opbg.net; 5 Université Paris Cité Sorbonne, 75006 Paris, France; dpayen1234@gmail.com; 6 Recherche Service Maladies Infectieuses, CHU de Nice, 06200 Nice, France

**Keywords:** pediatric septic shock, immune modulation, cytokine storm, multiple-organ dysfunction syndrome, immune dysfunction, immune paralysis

## Abstract

Pediatric sepsis presents a unique clinical challenge due to the distinct characteristics of the developing immune system. The immune response in children differs significantly from that in adults, exhibiting a unique combination of resistance, disease tolerance, and resilience. These factors influence the clinical presentation and prognosis of pediatric patients with sepsis. Over the past few years, various studies have explored the role of immunomodulatory therapies in managing sepsis, including the use of immunoglobulins, corticosteroids, monoclonal antibodies, and immunostimulatory treatments. However, the heterogeneity of the clinical presentations and individual responses makes it difficult to identify universally effective treatments. Recent research has highlighted the importance of a personalized approach based on specific biomarkers and patient phenotyping. Extracorporeal blood purification techniques have emerged as promising strategies for the modulation of hyperinflammation. However, strong evidence supporting their routine use in pediatric sepsis is lacking. This review provides a comprehensive overview of the current knowledge of the immune response in pediatric sepsis and discusses the main immunomodulatory strategies and future perspectives for personalized therapy. A deeper understanding of the immunological differences between children and adults could improve the prognosis and treatment efficacy, paving the way for new approaches to pediatric sepsis management.

## 1. Background

The complexity of sepsis immunobiology explains the failure of randomized controlled trials to test immunomodulatory therapies for sepsis over the last 10 years [1,2].

The host response to sepsis is characterized by concurrent hyperinflammation and immunosuppression, with highly heterogeneous clinical presentations [2,3]. The incomplete understanding of the underlying pathophysiology continues to slow down therapeutic advances [1,2]. Both the severity of the host’s dysregulated response and the trajectory of the process over time exhibit extreme variability [1,2]. Hyperinflammation is driven by the uncontrolled activity of pro-inflammatory effector mechanisms involving activated leukocytes and endothelial cells, along with the overwhelming presence of inflammatory mediators [1]. This process can lead to collateral damage (immunopathology) and contribute to sepsis-induced organ failure [1]. Furthermore, it is followed by an immunosuppressive profile, which increases the risk of nosocomial infections and viral reactivation [1,2,3].

This scenario is even more complex in children because of the development of their immune systems. The Phoenix definition of 2024 has acknowledged sepsis in children as a life-threatening infection [4,5,6], aligning with the Sepsis-3 definition [7] but adapting it to reflect the distinct immunobiology of critically ill children. However, to understand the rationale behind therapies, it is important to recognize the key differences that distinguish the host response in adults from that in children. The aim of this review is to provide, based on the existing literature, a description of the main characteristics of immunobiology in children compared to adults. Moreover, for the first time, to the best of our knowledge, we offer a comprehensive overview of the main immunomodulatory therapies currently in use and the existing evidence in this field.

## 2. Host Response in Children with Sepsis and Differences from Adult Populations

**Figure 1:**
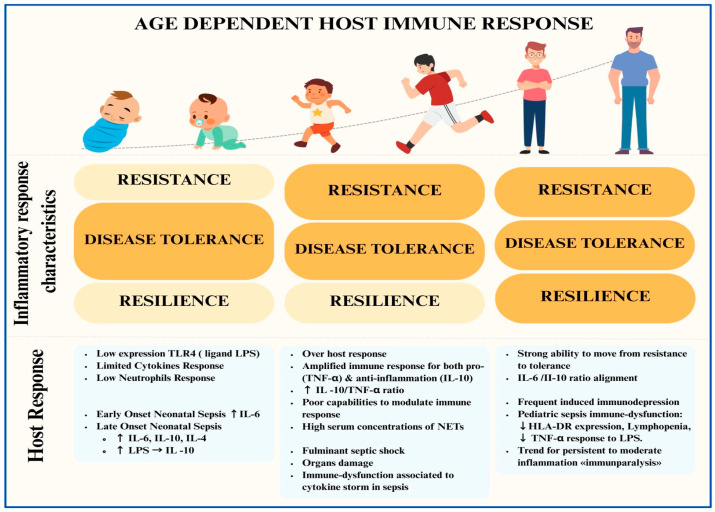
Graphical representation of sepsis immunobiology and host response during childhood development. **TLR** = Toll-like receptor; **LPS** = lipopolysaccharide; **IL-4** = interleukin 4; **IL-6** = interleukin 6; **IL-10** = interleukin 10; **TNF-α** = tumor necrosis factor alpha; **HLA-DR** = human leukocyte antigen—DR isotype; **NETs** = neutrophil extracellular traps.

Beyond the paradigms of hyperinflammation and immunosuppression, a recent review of the immunobiology of sepsis has extended the link between the immunological and metabolic phases. The initial phase aims to eliminate the pathogen (resistance), which is followed by a metabolic shift (catabolism) associated with an immunodepression profile that limits the host response-induced tissue damage (immunopathology) (disease tolerance). The third phase covers tissue repair (resilience), with the restoration of metabolic and immune function [8]. In the developing immune systems of children, resistance, disease tolerance, and resilience seem to have different capabilities according to their development as compared to adults. This maturation shapes specific patterns in the host response compared with adults. Preclinical models and clinical studies have confirmed that age and a history of microbial exposure can significantly influence immune biology [9] (Figure 1).

Compared with adults, newborns show diminished Toll-like receptor (TLR)-induced responses and reduced pro-inflammatory cytokine production [10]. Preclinical models have shown that the ability to produce cytokines in response to LPS stimulation increases with age [10]. Several neonatal immune cell types can directly regulate aberrant inflammation and promote tolerance. Newborns can produce and secrete cytokines and express markers of resident memory phenotypes [9]. Some authors have noted a significant difference in cytokine responses between early-onset sepsis (EOS) and late-onset sepsis (LOS), suggesting that the postnatal age may influence the disease [11]. Although both EOS and LOS patients showed a significant increase in serum IL-6 levels, only LOS patients released anti-inflammatory cytokines such as IL-10 and IL-4 [12].

During immune system development, a transition in T cell populations is observed. The predominant regulatory T cell populations in newborns shift to pro-inflammatory T cells in older children [13]. These T cells respond to antigens that may be under the control of IL-8 upregulation [13]. Similarly, the CD4 T helper (Th) cell population shifts from a Th2 phenotype (anti-inflammatory) to a Th1 phenotype (pro-inflammation), producing interferon-γ [13].

The neutrophils of newborns maintain low expression of TLR4, the ligand of LPS, on the surfaces of Gram-negative bacteria [13]. The expression of signal transducers immediately downstream of TLRs is also low in newborns, limiting cytokine responses and neutrophil chemotaxis [13]. However, there is evidence that pediatric patients with sepsis have significantly higher serum concentrations of neutrophil extracellular traps (NETs) than adults, and the severity of pediatric sepsis is positively correlated with the level of NETs [14,15,16].

**Figure 2:**
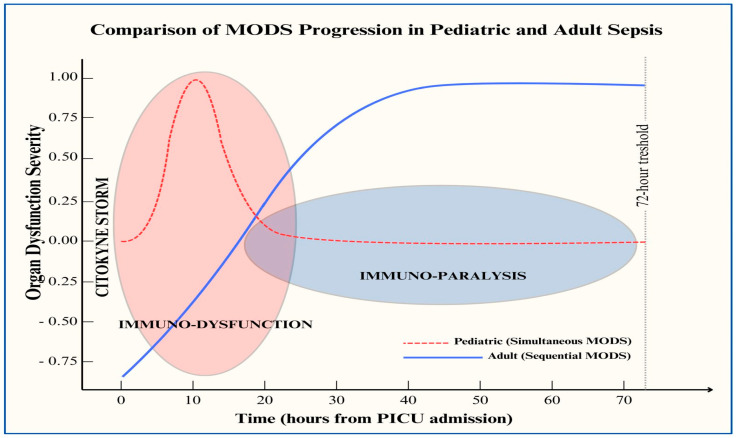
Comparative diagram of multiple-organ dysfunction syndrome trajectory in pediatric and adult populations with sepsis. **MODS** = multiple-organ dysfunction syndrome. The red ellipse in the image represents the exacerbated immune response in the pediatric host, associated with early (within 72 h) organ dysfunction and immunosuppression [12,13,14,15], following a simultaneous model (red dashed line) [13,14]. In contrast, the blue area represents the later-onset immune paralysis observed in the adult host after the cytokine storm, associated with MODS, following a sequential model (solid blue line) [13,14].

Differences in the innate and adaptive immune responses between childhood and adulthood may explain the specificity of sepsis in children [17]. An imbalance in inflammatory and compensatory anti-inflammatory responses in children may lead to septic shock and multiple-organ dysfunction syndrome (MODS) more frequently than in adults. Macrophages from young children produce greater amounts of both pro-inflammatory TNF-α and anti-inflammatory IL-10 than those from adults, and the resulting IL-10/TNF ratio is higher in children [17]. Using an ex vivo model of children’s macrophages stimulated by LPS, a 15-fold higher IL-10/TNF-α ratio than that in adults was observed [18,19]. Similarly to adults, clinical studies have demonstrated that higher initial cytokine responses in children are followed by impairments in innate immunity, as assessed by decreased monocyte HLA-DR expression [17]. Immunological tests showed that immunosuppression occurred as early as at the first 72 h in a cohort of children with severe sepsis and septic shock [20,21]. These findings strongly support previous reports indicating that children with sepsis exhibit a different pattern of MODS than adults (simultaneous vs. sequential) (Figure 2) [18,19]. These observations fit well with epidemiological data showing a higher rate of mortality within the first 72 h after PICU admission than in adults [22,23,24,25].

## 3. Management of Pediatric Septic Shock: Exploring New Perspectives Beyond Standard Care

Despite advancements in clinical management, such as early identification, appropriate resuscitation, and timely antibiotic therapy, the mortality rate associated with pediatric sepsis remains high. While adjuvant therapies for pediatric septic shock have been proposed, few demonstrations of their clinical benefits have been reported [26].

**Figure 3:**
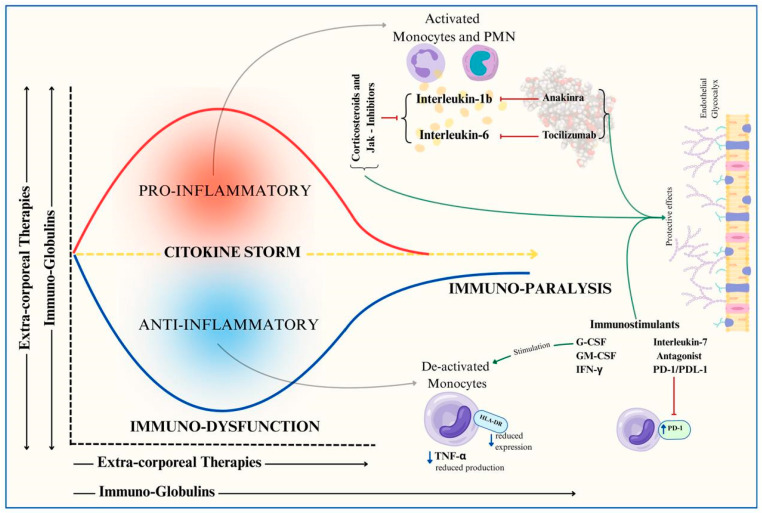
Graphical representation of possible immunomodulatory strategies in pediatric sepsis according to the sepsis trajectory. G-CSF = granulocyte colony-stimulating factor; GM-CSF = granulocyte-macrophage colony-stimulating factor; IFN-y = interferon gamma; **TNF-α** = tumor necrosis factor alpha; **HLA-DR** = human leukocyte antigen—DR isotype.

In recent decades, immunomodulation during septic shock has been extensively tested [27], targeting different inflammatory pathways in sepsis. Figure 3 provides a graphical representation of the possible immunomodulatory strategies in sepsis.

1. Cytokine modulation: Techniques such as extracorporeal blood purification therapies or the administration of immunoglobulins aim to control the overwhelming cytokine response.
2. Targeted immunomodulation: Selective drugs such as monoclonal antibodies block key mediators of septic shock.
3. Immune stimulation: Strategies to counteract immune paralysis through immunostimulatory therapies.

Targeting inflammatory pathways in sepsis is of increasing interest for the management of microcirculation alterations in sepsis. While the role of endothelial damage in pediatric inflammatory diseases is increasingly being recognized, the complex interplay between endothelial injury, the immune response, and therapeutic immunomodulation remains underexplored [28]. Endothelial damage, particularly involving the glycocalyx, initiates a cascade that includes the release of inflammatory mediators and immune cell recruitment, further perpetuating vascular injury. This bidirectional relationship contributes to microcirculatory dysfunction and increased microvascular permeability [29], which are hallmarks of severe pediatric conditions such as sepsis and multisystem inflammatory syndromes. Moreover, disruption of the endothelial glycocalyx not only compromises the vascular barrier but also alters cell signaling and leukocyte adhesion, exacerbating inflammation. Current evidence suggests that treatments aimed at restoring endothelial integrity, such as corticosteroids, anticoagulants, and biologics, play a crucial role in stabilizing the vascular barrier [30]. Therefore, future research and clinical guidelines should focus on how various therapies modulate endothelial function, with an emphasis on preserving or restoring vascular homeostasis in pediatric patients.

Although none of these approaches have demonstrated clear outcome benefits in sepsis, experts agree that this failure of clinical trials can be explained. Firstly, sepsis is a syndrome with multiple expressions, accounting for the great heterogeneity among patients [1,2]. Secondly, genetic susceptibility varies greatly within individuals and may change over time with environmental exposure, nutritional habits, and physical activity. Thirdly, age, comorbidities, chronic treatment, and pre-sepsis immunodepression may modify the host response. Different combinations of these features may naturally lead to distinct phenotypes with varying outcomes and responses to specific “trait-based” treatments [31].

Future clinical trial designs should better select the enrolled patients using parameters that reflect both clinical and biological characteristics, seeking to cluster patients more homogenously. This crucial step necessitates the use of validated biomarkers, especially those reflecting the major sub-endotypes based on molecular mechanisms.

As a consequence, the clinical strategy for therapies will be based on integrated inflammatory parameters such as coagulation, the longitudinal immune response, and cellular metabolic markers [32]. Selecting these parameters requires a deep understanding of the immunobiology, allowing one to intervene with a more personalized approach. Among these, adaptive interactions with immune and metabolic cellular changes lead to better results in sepsis.

## 4. Immunomodulation in Pediatric Septic Shock: Current Evidence

Evidence regarding immunomodulatory therapies for pediatric sepsis is limited. There is a lack of interventional studies conducted in an adequate pediatric population, and the validation and generalizability of the observational studies described need to be further explored.

The present authors, supported by two external librarians, electronically searched the literature using a combination of key medical terms related to immunomodulation AND septic shock; immunoglobulins AND septic shock; corticosteroids AND septic shock; monoclonal antibodies AND septic shock; immune stimulation AND septic shock; and extracorporeal blood purification therapies AND septic shock. The search was limited to pediatric patients (aged less than 18 years) and included randomized control trials (if available).

### 4.1. Immunoglobulins

**Figure 4:**
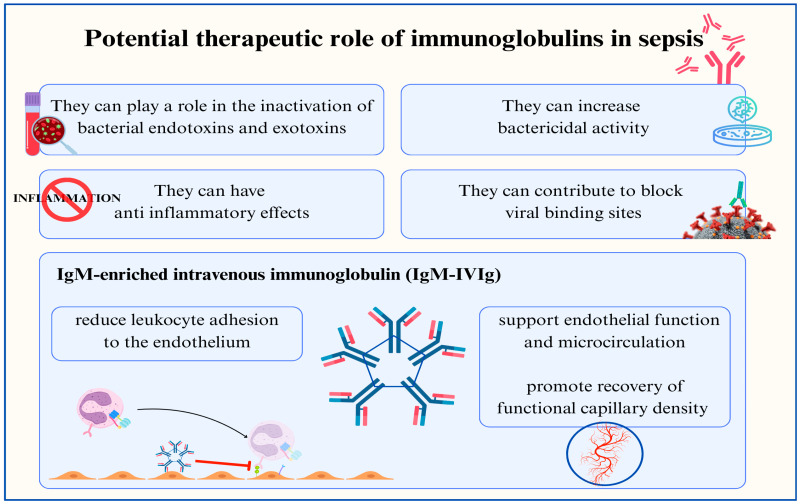
Graphical description of potential adjuvant mechanisms of immunoglobulins (IVIg) in sepsis, including IgM-enriched intravenous immunoglobulin (IgM-IVIg).

Immunoglobulins have been used for decades to treat several infectious diseases and immunological disorders. Intravenous immunoglobulin (IVIG) could be a valuable adjunctive therapy in sepsis for several reasons: it can help to inactivate bacterial endotoxins and exotoxins, blocks viral binding sites, enhances bactericidal activity, and exhibits anti-inflammatory effects [33] (Figure 4).

The international Guidelines on the Management of Pediatric Septic Shock and Sepsis-Associated Organ Dysfunction do not support the routine use of IVIG in children with sepsis or septic shock. However, emerging evidence suggests that some patients may benefit from this treatment [34]. These patients include children with toxic shock syndrome (TSS), necrotizing fasciitis, or primary immunodeficiencies or immunocompromised children with low levels of immunoglobulins [34]. Additionally, the absence of specific immunoglobulins in newborns during the first few months of life is one reason for which the administration of IVIG during this period could theoretically be highly beneficial [35].

A recent retrospective cohort study conducted by Huang et al. (2023) involved 304 hospitalized children with septic shock in the PICU of the Children’s Hospital of Chongqing Medical University [36]. Of these, 29.3% received intravenous immunoglobulin (IVIG). This group showed a greater need for continuous renal replacement therapy (CRRT) (43% vs. 24%, *p* = 0.001), a longer duration of mechanical ventilation (6 days vs. 3, *p* = 0.002), a longer length of stay in the pediatric intensive care unit (PICU) (7 days vs. 4, *p* = 0.002), and a longer hospital stay (18 vs. 11 days, *p* = 0.001). Survival at 28 days showed better results in the IVIG group (*p* = 0.033), while the in-hospital mortality did not exhibit significant differences between the two groups, although it was lower once again in the IVIG group. After propensity score matching, no significant differences were found in terms of CRRT, the mechanical ventilation duration, the PICU and hospital LOS, in-hospital mortality, and 28-day survival [36].

The aforementioned guidelines highlight that the administration of immunoglobulin M- and A-enriched polyclonal IVIG could be useful in managing septic shock [34]. Several adult-based studies have investigated this aspect, with noteworthy results [37,38,39,40]. The few reported pediatric studies on this topic suggest the potential benefit of using IgM-enriched immunoglobulins. In their systematic review and meta-analysis, Pan et al. (2023) emphasized the role of IVIG in reducing mortality and the in-hospital length of stay (LOS) (mainly including adult-based studies), stressing that IgM-enriched IVIG can reduce neonatal sepsis mortality with the efficacy of normal IVIG in adults [41].

A prospective study conducted by El-Nawaway et al. (2005) [42] at the PICU of Alexandria University Children’s Hospital showed promising results. Their study involved 100 children aged 1–24 years with sepsis, divided into two groups. Group I received traditional therapy plus IVIG (polyclonal IgM-enriched immunoglobulins), whereas Group II received only the traditional treatment. The results showed a significantly greater number of discharged patients in Group I than in Group II (36 vs. 22, *p* = 0.0046) and lower mortality rates in Group I (28% vs. 56%). Other findings included a lower tendency for Group I patients to develop complications in the PICU (8% vs. 32%, *p* = 0.0027), particularly disseminated intravascular coagulation (DIC). Additionally, Group I had a shorter LOS compared to Group II (6 vs. 9 days), despite presenting with more severe clinical conditions based on the PRISM III score at admission [42].

A retrospective study (the PIGMENT study), published in 2020, focused on the duration of treatment. Conducted in Eskisehir Osmangazi University Medical Faculty Hospital, the study included 254 children with sepsis, septic shock, and multi-organ failure, admitted to the PICU from January 2010 to December 2017. Of these, 104 patients received treatment for three days, while 150 received treatment for five days. The mortality rate was lower in the group of patients who underwent 5-day-long treatment (20.6% vs. 40.3%; R:0.51, 95% CI 0.34–0.75; *p* < 0.001) [43]. Table 1 provides a summary of the main clinical studies on the use of immunoglobulins (IVIG) and IgM-enriched IVIG in pediatric sepsis.

### 4.2. Immunoglobulins and Toxic Shock Syndrome

As mentioned previously, the use of IVIG in children with TSS is a distinct consideration. TSS is a severe illness associated with infections caused by *Staphylococcus aureus* and *Streptococcus pyogenes*. Its pathogenesis involves massive cytokine release in response to bacterial toxins [44]. Wilkins et al. [45] outlined the steps in managing TSS, including IVIG and clindamycin as adjunctive therapies. IVIG appears to offer benefits in terms of antigen recognition, the activation of the innate immune system, and the neutralization of super-antigen toxin activity [45]. A 2018 systematic review and meta-analysis, although primarily focused on adults, found a reduction in mortality among patients with streptococcal TSS treated with IVIG in combination with clindamycin [46]. Interestingly, in the only pediatric study included, none of the patients treated with IVIG died, while the overall mortality rate was 16% [47].

Evidence of the beneficial effects of immunoglobulins in critically ill children with toxic shock syndrome is contradictory. A pediatric retrospective analysis showed promising results: 94% of the patients received IVIG and no deaths were recorded [48]. A multicenter retrospective cohort study involving children with streptococcal TSS reported a mortality rate of 4.2%, with no statistically significant differences between the IVIG-treated and control groups [49].

### 4.3. Corticosteroids

**Figure 5:**
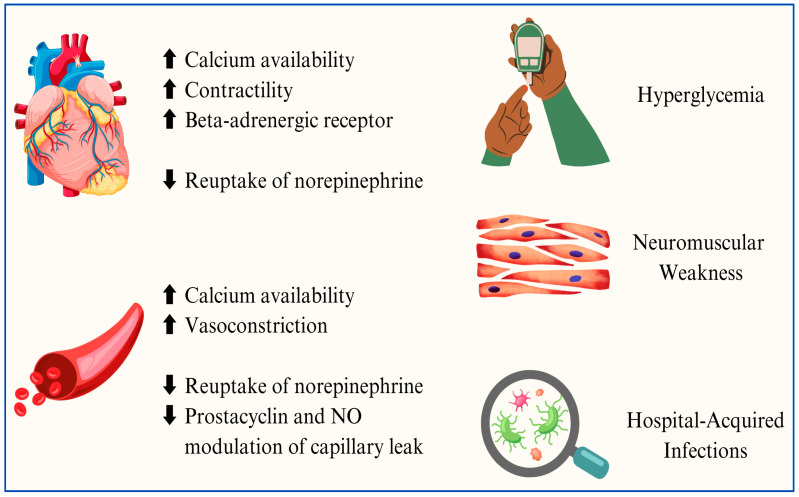
Graphical description of the potential beneficial and adverse effects of corticosteroids in sepsis.

Cortisol plays different roles in septic shock: it directly decreases the reuptake of norepinephrine, enhances calcium availability in myocardial and vascular smooth muscle cells, promotes myocardial contractility and vasoconstriction, and inhibits prostacyclin and endogenous nitric oxide production, resulting in increased vascular tone, the modulation of capillary leaks, and the augmentation of the beta-adrenergic receptor in the heart [34]. On the other hand, adverse effects caused by corticosteroid therapy include hyperglycemia, catabolism-related diffuse neuromuscular weakness, and hospital-acquired infections [34] (Figure 5).

The use of corticosteroids as an adjunctive therapy for septic shock remains a topic of debate. The current Surviving Sepsis guidelines recommend hydrocortisone only for fluid-refractory, catecholamine-resistant, and suspected/proven adrenal insufficiency [34].

In adults, Annane et al. demonstrated that a 7-day treatment with low doses of hydrocortisone and fludrocortisone significantly reduced the mortality risk in patients with septic shock and relative adrenal insufficiency, without increasing the number of adverse events (hazard ratio 0.67; 95% confidence interval 0.47–0.95; *p* = 0.02) [50]. Conversely, another RCT involving 3800 adults with septic shock on mechanical ventilation found that continuous hydrocortisone infusion did not reduce 90-day mortality compared with a placebo (odds ratio 0.95; 95% confidence interval [CI] 0.82 to 1.10; *p* = 0.50) [51].

However, evidence in critically ill children with septic shock is limited. In a randomized control trial, Valoor et al. reported a trend toward earlier shock reversal (median 49.5 vs. 70 h, *p* = 0.65) and lower inotrope requirements (median inotrope score 20 vs. 50, *p* = 0.15) in hydrocortisone-treated patients, although the differences were not statistically significant. The mortality rates were similar between the groups (*p* = 1.0) [52]. El-Nawawy et al. found a significantly shorter shock reversal time in patients receiving corticosteroids at the start of treatment compared with those receiving them later (*p* = 0.046), although the mortality rates were similar (*p* = 0.734) [53]. Menon et al. analyzed 49 pediatric patients and found no significant differences in the time on vasopressors (*p* = 0.65), days of mechanical ventilation (*p* = 0.37), or PICU length of stay (*p* = 0.48) [54]. Alkhalf et al. observed a 42% lower risk of prolonged PICU stays in patients receiving steroids, but no difference in mortality (*p* = 0.492) [55].

Recent evidence suggests a need for a personalized approach that acknowledges the variable responses to steroids. Alder et al. found that children with different glucocorticoid receptor (GCR) and cortisol concentrations may respond differently to corticosteroids. In their study, pediatric patients with complicated septic shock (defined as two or more organ failures by day 7 or death by day 28) had lower GCR expression and higher cortisol levels. This subgroup showed a 75% rate of complicated outcomes, compared with 13–33% in other GCR and cortisol combinations (*p* < 0.05) [56].

Wong et al. developed a gene expression panel that segregates children with acute septic shock into endotypes based on the immune response and GCR signaling. Children with endotype A, characterized by the underactivation of adaptive immunity and GCR signaling, had increased mortality when treated with corticosteroids (OR = 4.1; CI95 = 1.4–12.0; *p* = 0.011) [57]. Approximately one-third of the children with septic shock exhibited a change in endotype assignment during the first 3 days, with those persisting as endotype A at the highest risk for poor outcomes. This evolving understanding highlights the importance of individualized treatment strategies in managing pediatric septic shock with corticosteroids [57,58]. Table 2 provides a summary of the main clinical studies on the use of corticosteroids for pediatric sepsis.

### 4.4. Monoclonal Antibodies

**Human IL-1 receptor antagonist (rhIL-1ra)**. In 1994, Fisher et al. conducted a study that found no significant survival benefit from anakinra in sepsis (*p* = 0.22) [59]. However, a secondary analysis showed a dose-related increase in the survival time among patients with organ dysfunction [60]. These data were re-analyzed by Shakoory et al. in 2016, focusing on patients with hepatobiliary dysfunction and disseminated intravascular coagulation, which are features of macrophage activation syndrome (MAS), and found that anakinra treatment improved the 28-day survival rate (HR = 0.28, *p* = 0.0071) [60]. A trial by Opal et al. in 1997 was halted after an interim analysis revealed no significant difference in mortality at 28 days between the case (33.1%) and control (36.4%, *p* = 0.36) groups [61].

A recent narrative review by Manchikalapati et al. in 2023 highlighted the need for randomized controlled trials (RCTs) to study the potential role of anti-IL-1 in pediatric sepsis [62]. The review discussed five studies involving anakinra in children with secondary hemophagocytic lymphohistiocytosis (HLH), 72 of whom also had sepsis [63,64,65,66]. However, the results were inconclusive. Currently, there are two ongoing studies investigating the use of anakinra in sepsis. The first was ImmunoSep (personalized immunotherapy in sepsis), a double-blind, placebo-controlled, randomized phase II clinical trial (NCT04990232), which included 280 patients [67]. The second is a prospective, multicenter, double-blind, placebo-controlled clinical trial involving children with sepsis (NCT05267821) [68]. Table 3 provides a summary of the main clinical studies on the use of human IL-1 receptor antagonists (rhIL-1ra) in pediatric sepsis.

**Anti-interleukin (IL)-6 antibodies**: A systematic review in 2023 summarized its efficacy and safety in sepsis and SARS-CoV-2 infection [69]. Tocilizumab was associated with reduced 28-day mortality (RR 0.88, 95% CI 0.81–0.94), although its impact on 60-day mortality remains uncertain [70]. It also showed a favorable safety profile, with minimal differences in adverse effects compared with traditional treatments [71]. Regarding the use of tocilizumab in sepsis, only two papers have been published, involving a total of six adult patients, where the antibody showed promising results [72].

**Janus kinase (JAK) inhibitors**: Song et al.’s 2023 systematic review and meta-analysis explored the role of baricitinib in COVID-19 patients [73]. The study found that baricitinib reduced mortality (OR = 0.61, *p* = 0.008) and the need for mechanical ventilation (OR = 0.57, *p* = 0.002) [73]. There was no significant difference in the in-hospital length of stay or adverse effects between the baricitinib and control groups [73]. Currently, no data are available regarding the use of baricitinib in patients with sepsis or pediatric septic shock.

### 4.5. Immunostimulation

The dysregulated immune system in sepsis and septic shock may induce a phase of depression of the immune system (called “immunoparalysis”), inactivating immune cells and contributing to mortality and morbidity due to secondary infections [74].

Recently, criteria to detect signs of immune paralysis in children were established in the PODIUM consensus [75], potentially identifying those who might benefit from immunostimulatory therapies. These are different therapeutic approaches that aim to impact different molecular pathways, including cytokines, immune cells, and growth factors. Pediatric studies are a minority, but the trials are growing in number.

**Granulocyte Colony-Stimulating Factor (G-CSF) and Granulocyte-Macrophage Colony-Stimulating Factor (GM-CSF)**. G-CSF and GM-CSF are hematopoietic stimulators that enhance the production, migration, survival, and activity of neutrophils, monocytes, and other immune cells. Because of these properties, they have been explored as supportive therapies for patients with sepsis [74]. A meta-analysis of adult patients with septic shock indicated that, while G-CSF and GM-CSF improved immune cell function, they did not significantly affect overall mortality at 14 or 28 days (16.6% vs. 17.6%; *p* = 0.44) or in-hospital mortality. However, they were associated with the greater resolution of infections (29.4% vs. 21.8%; *p* = 0.002) [76]. This analysis suggests that these drugs should not be routinely administered in sepsis [76], particularly without immune monitoring.

In pediatric patients with MODS and immune paralysis, GM-CSF prevented secondary infections and helped to recover immune system function more quickly than in the placebo group [77]. A retrospective study of 109 pediatric sepsis patients found that those receiving immunomodulatory therapy, including G-CSF, required more frequent PICU admission and invasive ventilation. However, they had shorter ventilator-free and PICU-free periods [78]. Studies on neonates with sepsis have demonstrated that G-CSF and GM-CSF could restore immune cell function, including increasing HLA-DR expression and monocyte numbers [79,80].

The literature remains divided on the use of G-CSF and GM-CSF in sepsis treatment, particularly because of the potential for adverse effects such as organ failure [81]. Based on a Cochrane meta-analysis [82], which showed no benefit of GM-CSF in newborns and infants who were not selected based on the presence of neutropenia, the need for personalized treatment approaches when using immune-stimulatory therapies in sepsis has been highlighted [82].

**Interleukin-7**. One of the main functions of IL-7 is to prevent a decrease in lymphocytes by stimulating their survival and proliferation, particularly CD4+ and CD8+ T cells. This action helps to increase pathogen clearance and protects against secondary infections by recruiting neutrophils to the peripheral tissue. In a study of adult septic patients after IL-7 treatment, lymphocytes showed increased numbers (*p* < 0.05) and glucose utilization (*p* < 0.05) via the better functioning of the mTOR pathway (*p* < 0.05), indicating their ability to reverse metabolic alterations [83]. The IRIS-7 trial, which was a randomized double-blind trial, investigated CYT107 (recombinant human IL-7) and reported an increase in lymphocyte numbers (*p* < 0.001), particularly CD4+ and CD8+ T cells, owing to its anti-apoptotic effect and impact on cell expansion. Although not powered for outcome evaluation, the trial did not affect the 28-day mortality rate (*p* = 0.687) [84]. Currently, there are no studies available on the efficacy of recombinant human IL-7 in pediatric septic shock.

**Interferon-γ**. Several studies have focused on the effects of exogenous IFN-γ on immunoparalysis in patients with sepsis. IFN-γ increases HLA-DR expression in circulating monocytes, enhances their activation, and stimulates neutrophil function to eliminate the microbes. It can only be used after the diagnosis of immunoparalysis; if so, treatment must be carefully monitored. Consequently, its early use during a cytokine storm is contraindicated, as recently reported [85]. A pioneering study involved nine adult septic patients with impaired HLA-DR expression, which increased after a subcutaneous injection of IFN-γ (100 µg). In parallel, INF-γ enhanced TNF production in vitro. Eight patients recovered after the treatment [86]. A remarkable study in 18 healthy volunteers induced a reduction in the monocyte expression of HLA-DR via the injection of small doses of LPS. Only delayed exogenous INF-γ administration prevented the reduction in TNF levels (*p* = 0.01), lowered the IL-10 levels, and restored normal HLA-DR expression (*p* = 0.02). GM-CSF treatment showed a similar trend but this did not reach statistical significance [87]. A tri-centric case series evaluated IFN-γ treatment in patients with septic shock: cohort 1 received IFN-γ based on criteria such as ICU hospitalization > 7 days, the presence of secondary infections despite antibiotics, and persistently low HLA-DR expression on immune cells. The first group showed a significant increase in monocyte HLA-DR expression and negative culture after IFN-γ treatment and was discharged from the ICU. Cohort 2 began INF-γ treatment when their norepinephrine doses were halved and showed a similar improvement in HLA-DR expression. Additionally, a pediatric liver transplant case with septic shock followed by deep immunoparalysis and untreatable infections recovered after IFN-γ therapy with a maintained protocol of anti-rejection drugs [88].

In premature newborns, who often have an immature immune system that deteriorates during sepsis, IFN-γ administration has been investigated to restore normal immune function. The observed increase in TNF-α and IL-6 levels was associated with a decrease in IL-10 levels. The pathogen receptors for bacteria in immune cells increase dramatically, and phagocytosis returned to normal [89]. However, the lack of pediatric trials suggests a need for further investigation. The general approach is to identify patient subgroups and test immunostimulation with a unique drug, or those in combination, in the late phase of sepsis [85].

**Immune checkpoints**. Some studies have explored the use of PD-1 and PDL-1 antagonists, known as “immune checkpoints”, to counteract immune cell apoptosis and paralysis during sepsis and restore immune function [85]. PD-1 is mainly expressed on T cells, whereas PDL-1 appears on antigen-presenting cells, and its activation inhibits T cell function and significantly reduces IL-2 and IFN-γ production [85,90].

A clinical trial involving adult septic patients administered anti-PDL-1 antibodies demonstrated a safe increase in HLA-DR expression. However, more studies are needed to understand its effects on the prevention of secondary infections and the overall survival rate [91]. A study involving 43 adult septic patients showed benefits when anti-PD-1 and anti-PDL-1 drugs were administered, including increased cytokine levels (e.g., IL-2; *p* < 0.01), reduced immune cell apoptosis (*p* < 0.01), and improved IFN-γ secretion (*p* < 0.01) [92].

**Others—Glutathione Peroxidase 4 (GPX4)**: A recent study in children investigated GPX4, which plays a crucial role in sepsis diagnosis and is associated with the disruption of oxidative balance, lipid peroxide accumulation, and ferroptosis. The loss of GPX4 function is linked to organ failure in sepsis and worsens the prognosis by damaging mitochondria. This study also highlighted an imbalance in the immune system during sepsis, potentially due to altered GPX4 function [93]. Table 4 provides a summary of the main clinical studies on the use of immunostimulation therapy in pediatric sepsis.

### 4.6. Extracorporeal Blood Purification Techniques in Pediatric Septic Shock

**Figure 6:**
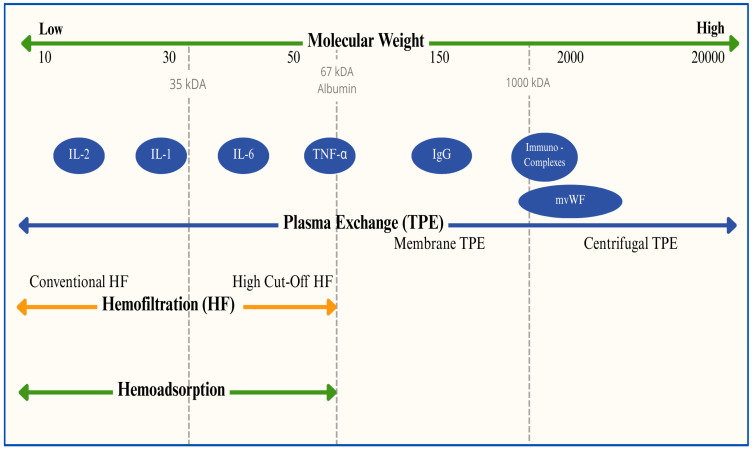
Various extracorporeal blood purification techniques based on their target mediators in sepsis. **kDa** = kilodalton; **IL-2** = interleukin 2; **IL-1** = interleukin 1; **IL-6** = interleukin 6; **TNF-α** = tumor necrosis factor alpha; **IgG** = immunoglobulin G; **mWF** = Von Willebrand multimers; **TPE** = therapeutic plasma exchange; **HF** = hemofiltration.

Renal replacement therapies (RRTs) are used to support or replace kidney function in critically ill patients with acute kidney injury (AKI). These therapies help to maintain the fluid and chemical balance while eliminating waste products from the body. RRT is crucial for the management of AKI, including sepsis-associated AKI [94]. The “peak concentration” hypothesis, introduced in 2003, proposes that partially clearing cytokines from the bloodstream during the early stages of sepsis could lower their peak levels and help to regulate the inflammatory response [95]. The potential advantages of extracorporeal blood purification therapies have gained further attention with the recognition of “organ cross-talk” in sepsis, where dysfunction in one organ can negatively impact others, particularly in cases of multiple-organ dysfunction syndrome (MODS). This approach is based on the idea that malfunctioning organs remain perfused with blood, making this a viable target for therapeutic interventions [96,97,98,99]. Figure 6 provides a graphical representation of extracorporeal blood purification techniques according to removal target among mediators of the sepsis process. 

**Hemofilters with adsorption capacities**. AN69ST is a hemofilter that can be mounted on normal CRRT devices with a polyacrylonitrile membrane on which a surface treatment (ST) has been applied by grafting a biocompatible polymer named polyethyleneimine (PEI). In a prospective cohort study, the use of AN69ST as a hemofilter in patients showed a trend of decreasing pro-inflammatory and anti-inflammatory cytokines, and membrane adsorption emerged as the main cytokine clearance mechanism [100]. The subsequent AN69 Oxiris^®^ version is characterized by a three-layer structure, and the second layer consists of PEI at concentrations three-fold higher than those in the AN69ST filter, enabling the biologically active PEI surface to adsorb endotoxins. Recently, Morin reported the use of Oxiris^®^ in a single-center prospective observational study. The cohort included pediatric patients with vasoplegic shock and acute kidney injury (seven of 11 patients affected by septic shock). The authors reported a 50%reduction in inotropic support within 24 h (considered a success in the treatment) in 5 of 11 patients (four of seven with septic shock) [101].

**Polymyxin B (PMX-B)** is a basic cyclic polypeptide that alters the permeability of the membranes of Gram-negative bacteria. PMX-B immobilized on polystyrene fibers was developed as an extracorporeal clearance system to remove endotoxins, with hemoadsorption cycles of 2 h for 2 days [101]. Interestingly, PMX-05R and PMX-01R are Toraymixin^®^ cartridges (Toray Medical Co., Houston, TX, USA) with safe priming volumes in newborns and children [102]. The results of two large multicenter RCTs demonstrated a non-significant increase in mortality and no improvement in organ failure with PMX-HP treatment compared with the conventional treatment of peritonitis-induced septic shock [103,104]. Two ancillary studies have reported the effects of PMX-HP on the plasma levels of 21 cytokines [105] and the actual effect on the mass of plasma LPS [106]. Surprisingly, none of these studies demonstrated any effect of PMX-HP treatment on the plasma cytokine levels or on the plasma mass of LPS (mass spectrometry). These results shed light on the significant reduction in LPS mass caused by PMX-HP. It is therefore possible that the rate of LPS release into the plasma dominates the extraction capacity of the membrane.

However, the EUPHRATES post hoc analysis highlighted that, in a subgroup of patients with an indirect endotoxin assay value between 0.6 and 0.9, an improvement in survival was found; this provides future opportunities for a new study with a more appropriate population [107,108,109].

Clinical experiences in newborns with septic shock who were successfully treated with hemoadsorption using a PMX-B-immobilized fiber column have been reported in Japan [110,111]. Moroshita et al. described their experience using blood purification with PMX-B in a 13-month-old child with sepsis due to *Pseudomonas aeruginosa*, with the resolution of the clinical picture [112]. Nanishi et al. reported their use of PMX-B in an adolescent with toxic shock syndrome, with a positive outcome, thus questioning the potential benefit of PMX-B for Gram-positive bacterial infections [113].

Polymyxin B hemoadsorption with PMX-05 cartridges was applied to 15 children with septic shock. The authors reported a trend towards an improvement in hemodynamics after two sessions of therapy, although the inotropic agent requirements did not change over time [114]. Recently, Saetang et al. described the use of PMX-20R (a modified polymyxin-B cartridge circuit for pediatric use) in six children and reported a significant reduction in the PELOD-2 and VIS scores after two sessions of therapy [115].

Regarding **plasma separation (PE)** techniques, plasmapheresis is a technique used for the separation of plasma from blood cells, and it can be performed by centrifugation with cell separation to reach ≥80% hematocrit or by membrane separation with porous fibers. The latter technique is less efficient because blood cell separation using a membrane system can be hindered when very high hematocrit values are reached. In addition, the upper cutoff of the TPE membrane is 1000 kDa, suggesting that molecules with higher molecular weights, such as Von Willerbrand multimers, cannot be removed [116].

Nguyen T. [117] showed an improvement in mortality at 28 days and in organ dysfunction scores in pediatric patients treated with PE. The trial was conducted exclusively in children with septic shock and thrombocytopenia-associated multiple-organ failure (TAMOF). The study conducted by the above-mentioned authors showed that children with septic shock presenting a clinical picture of TAMOF had reduced ADAMTS13 activity, and autopsies of non-survivors revealed microcirculation thromboses with von Willebrand factor-rich microaggregates [117]. Therefore, PE was indicated as a therapeutic priority in this subgroup of patients [117,118].

A recent systematic review by Lee highlighted that PE use may be associated with harmful effects in pediatric patients with sepsis and septic shock. However, this has not been confirmed in studies including pediatric patients with septic shock and a TAMOF phenotype [119].

**Adsorption columns** are new options for adsorbent membranes. They are characterized by a very large surface area (8,500 m^2^) compared to traditional membranes (1.5 m^2^), which could make them a very effective tool for hypercytokinemia, with potential therapeutic efficacy in sepsis [120]. Recently, hemoadsorption with new sorbents customized to adsorb cytokines and other inflammatory mediators has emerged as the most investigated and clinically established procedure in the clinical context of sepsis [121].

CytoSorb^®^ (Cytosorbents Europe GmbH, Berlin, Germany) is a hemocompatible cytokine adsorber, whose unique structure allows the generation of hydrophobic bonds with cytokines, which are adsorbed, while plasma proteins are filtered among micropores and returned to the systemic circulation [122]. In 2023, the first pediatric interventional study confirmed the benefit of Cytosorb^®^ in combination with CRRT in pediatric patients with septic shock, in terms of reducing the need for vasopressors and inotropes, as well as 28-day mortality, compared to a historical cohort of pediatric patients with septic shock treated only with CRRT [123]. In addition to a secondary analysis, the authors showed a significant reduction in IL-6 and IL-10 levels, with evidence that, at 24 h after the end of hemoadsorption, no rebound effect was observed, suggesting that the extracorporeal therapeutic effect could substantially affect immune homeostasis [124]. Furthermore, longitudinal measurements of monocyte HLA-DR expression in the enrolled population showed that hemoadsorption prevented the expected downregulation of HLA-DR [124].

Jafron^®^ cartridges (Guangdong, China) containing neutro-macroporous resin-adsorbing beads composed of a styrene–divinylbenzene copolymer have been used for the removal of a wide spectrum of molecules. HA330^®^ cartridges have also been used to treat children [125,126]. Jafron recently released two novel devices specifically dedicated to the care of the smallest numbers of patients, i.e., HA60 and BS80. The first is devoted to the care of septic children with a priming volume of 60 mL, and the second is focused on hyperbilirubinemia with a priming volume of 50 mL. The current literature on these materials is limited to non-indexed publications, but the evolution of this technology to miniaturized devices will certainly help to improve the available evidence in pediatric and neonatal settings [127].

## 5. Knowledge Gaps and Research Opportunities in Pediatric Sepsis

Despite existing evidence suggesting the role of immunomodulatory therapies in pediatric sepsis, the widespread translation of these results into clinical practice is still far from being realized. In particular, there is a general need to determine how to implement the personalization of therapy. The heterogeneity of the immune responses in children complicates the identification of effective targeted treatments. The current research also lacks robust biomarkers to stratify patients based on their immune status, leading to challenges in clinical decision-making. Table 5 provides a summary of some key points related to the knowledge gaps and research opportunities for immunomodulatory therapies in pediatric sepsis.

## 6. Conclusions

Pediatric sepsis is a complex condition that requires a deep understanding of the specific immunobiology of children to develop effective therapeutic strategies. Research has shown that the immune system in children differs significantly from that of adults. These characteristics not only influence disease progression but also affect the responses to immunomodulatory treatments.

Therapeutic approaches for pediatric sepsis are continuously evolving. Current strategies include the use of immunoglobulins, corticosteroids, monoclonal antibodies, and blood purification techniques. However, regarding pediatric intensive care, there is still a relatively small number of studies on immunomodulation in pediatric sepsis and septic shock, leading to recommendations that are based largely on expert opinions rather than on published data. In this context, the difficult transition in the science of intensive care medicine—from evidence-based medicine to personalized medicine—becomes particularly evident.

A crucial aspect of the future management of pediatric sepsis will be the development of diagnostic tests capable of identifying specific biomarkers to stratify patients (phenotype, sub-endotypes) and monitor the drug response, avoid iatrogenicity, and fully implement the concept of personalized treatment.

## Figures and Tables

**Table 1:** Summary of the main clinical studies on the use of immunoglobulins (IVIG) and IgM-enriched IVIG in pediatric sepsis. **PICU**: pediatric intensive care unit; **MV**: mechanical ventilation; **CRRT**: continuous renal replacement therapy; **IVIG**: intravenous immunoglobulin.

IVIG						
Reference	No. of Patients	Study Design	Study Period	Age	Outcomes	Mortality
Huang et al. (2023) [36]	304	Retrospective cohort study	1 January 2017–31 December 2021	7–144 months	Primary: in-hospital mortality Secondary: PICU duration of stay, length of hospital stay, requirement for MV and CRRT	No-IVIG group: 112 (52%); IVIG group: 38 (43%)
**IgM-enriched IVIG**						
Pan et al. (2023) [41]	6276	Systematic review and meta-analysis	Studies published up to 31 January 2023	Neonates and adults	Primary: mortality at end of follow-up period Secondary: length of hospital stay	Inconclusive regarding effect of IVIG in reducing mortality among neonates (RR: 0.93; 95% CI 0.81–1.05); IgM-rich IVIG showed a positive effect in the treatment of neonatal sepsis (RR 0.45; 95% CI: 0.25–0.80)
El-Nawaway et al. (2005) [42]	100	Prospective study	2022	1–24 months	To study differences between control group (standard treatment) and case group receiving polyclonal IVIG in addition	Controls had a smaller percentage of mortality at 14 (28%) vs. the control group at 28 (56%)
Abdullayev et al., PIGMENT study (2002) [43]	254	Retrospective study	January 2010–December 2017	1 month–18 years old	To evaluate clinical features and prognoses of children receiving IgM-enriched IVIG	Mortality rate was 28.7%; in particular, it was 40.3% (#42) for the 3-day treatment group and 20.6% (#31) for the 5-day treatment group (OR: 0.51; 95% CI 0.34–0.75)

**Table 2:** A summary of the main clinical studies on the use of corticosteroids for pediatric sepsis. Abbreviations:; **CI**: confidence interval; HR: hazard ratio; **OR**: odds ratio.

Corticosteroids						
Reference	No. of Patients	Study Design	Study Period	Age	Mortality N (%)	Dose and Type of Corticosteroids
Valoor et al. (2009) [52]	38	Open-label randomized pilot study	Subjects were enrolled within 30 min of the time that fluid refractory shock was diagnosed, and the time for shock reversal was calculated.	2 months–12 years	Control group: 7 (37%). Placebo group: 6 (32%).	Control group: intravenous hydrocortisone 5 mg/kg/day in four divided doses, followed by half the dose for a total duration of 7 days
El-Nawawy et al. (2017) [53]	96	Prospective interventional randomized clinical trial	30 day follow-up	1 month–4 years	Group C: deceased (30-day mortality) 14 (43.75%). Group D: deceased (30-day mortality) 20 (55.55%)	Group C: intravenous hydrocortisone 50 mg/m^2^/24 h with continuous infusion for 5 days from admission and weaning of the drug over 5 days Group D: corticosteroids in the third stage of therapy
Kusum Menon et al. (2017) [54]	101	Randomized, double-blind, placebo-controlled, multicentric trial	Screening period: July 2014–March 2016. The total number of recruitment months was 90 across all study sites, with the site-specific recruitment period ranging from 2 to 20 months.	Children from newborn to 17 years old inclusive	Placebo group: 3 (6%). Control group: 1 (2%). *p* = 0.61.	Control group: an initial intravenous bolus of 2 mg/kg hydrocortisone, followed by 1 mg/kg of hydrocortisone every 6 h until the patient met stability criteria for at least 12 h. Hydrocortisone dosing was then reduced to 1 mg/kg every 8 h until all vasoactive infusions had been discontinued for at least 12 h for a maximum of 7 days.
Alkhalaf H.A. et al. (2023) [55]	182	Retrospective cohort study	Study period: January 2016–December 2021	<14 years old	After adjusting for baseline characteristics, severity scores, and medical intervention, no statistical association was found between corticosteroid use and mortality (HR: 2.61; 95% CI 0.66–10.28).	Steroid regimen not specified
Alder et al. (2018) [56]	164	Prospective cohort study	28 days follow-up	<18 years old	Mortality, n (%): SIRS: 2 (12); sepsis: 0 (0); septic shock: 6 (8)	No steroid administration
Wong H.R. et al. (2015) [57]	Study subjects (n = 168) Separate cohort (n = 132)	Development and validation study, prospective cohort study (for the validation and outcome analysis phase)	28 days follow-up	0.2–7.3 years old	Derivation Cohort: Subclass A: 12 (21); Subclass B: 11 (10). Test Cohort: Subclass A: 11 (17); Subclass B: 4 (5). Adjunctive corticosteroids increased risk of mortality in subclass A (OR = 4.1; *p* = 0.011), but not in subclass B.	Steroid regimen not specified
Wong H.R. et al. (2018) [58]	375	Observational cohort study	28 days follow-up	≤10 years	28-day mortality, n (%): Endotype AA: 12 (16); Endotype AB: 10 (18); Endotype BB: 8 (5); Endotype BA: 1 (1).	Steroid regimen not specified

**Table 3:** A summary of the main clinical studies on the use of human IL-1 receptor antagonists (rhIL-1ra) in pediatric sepsis.

rhIL-1ra								
Reference	No. of Patients	Study Design	Study Period	Age	Clinical Presentation	Outcomes	Mortality	Further Results
Rajasekaran et al. (2014) [63]	8	Retrospective case series	1 January 2011–31 July 2012	8–21 years old	Patients with secondary HLH admitted to PICU	To study the role of anakinra in reducing systemic inflammation	1 (12.5%)	5 (62.5%) needed MV; 5 (62.5%) required vasoactive therapy; 1 (12.5%) needed RRT
Gregory et al. (2019) [64]	33	Retrospective electronic medical record review	2007–2017	27–186 months	Patients with both familial and secondary HLH	To study both in-hospital mortality and 1-year mortality	7 in-hospital deaths (21%); 1-year mortality was 27%.	48% received anakinra (42% of survivors and 71% of non-survivors)
Eloseily et al. (2020) [65]	44	Retrospective review	January 2008–December 2016	1–19 years old	Children with secondary HLH	To analyze the role of anakinra in the treatment of secondary HLH	12 (27%)	Early anakinra administration (<5 days of hospitalization) was associated with a reduction in mortality (*p* = 0.046).
Charlesworth et al. (2021) [66]	3	Case series	/	9, 11, and 17 years old	Severe secondary HLH/MAS	To report 3 cases of critically ill children who received IV anakinra	0	The study underlines the safety and efficacy of anakinra in patients with infection.

**Table 4:** A summary of the main clinical studies on the use of immunostimulation therapy in pediatric sepsis. **G-CSF**: granulocyte colony-stimulating factor; **GM-CSF**: granulocyte-macrophage colony-stimulating factor; **PICU**: pediatric intensive care unit; **HLA**: human leukocyte antigen; **IFN-γ**: interferon; **LPS**: lipopolysaccharide; **ELBW**: extremely low birth weight; **GPX4**: glutathione peroxidase 4.

G-CSF and GM-CSF						
Reference	No. of Patients	Study Design	Study Period	Age	Outcomes	Mortality/Results
Lee et al. (2021) [78]	109	Retrospective review	1 January 2010–31 October 2017	Children	PICU mortality, 28-day ventilator-free days (VFD), and intensive care unit-free days (IFD)	PICU mortality was not different between the 2 groups (20/54 [37.0%] vs. 11/55 [20.0%], *p* = 0.058)
Bilgin et al. (2001) [80]	60	RCT	January 1994–March 1995	Neonates	Assessing whether rhGM-CSF could reverse neutropenia and other hematologic parameters in septic neonates and improve neonatal survival, compared to conventional therapy in a control group	All neonates tolerated GM-CSF. Neutrophil numbers increased on day 7 after GM-CSF, compared with the conventionally treated group (8088 ± 2822/mm^3^ vs. 2757 ± 823/mm^3^) (*p* < 0.01). The mean platelet count was significantly higher on day 14 in the GM-CSF-group (266,867 ± 55,102/mm^3^ vs. 229,200 ± 52,317/mm^3^) (*p* < 0.01). Other hematologic parameters were similar between groups on day 28. Twenty-seven neonates in the rh-GMCSF group and 21 in the control group survived. The mortality rate in the rhGM-CSF group (10%) was significantly lower than in the conventionally treated group (30%) (*p* < 0.05).
Drossou-Agakidou et al. (2002) [79]	60	RCT	Follow-up during the study	Neonates	Assessing the increase in HLA-DR on monocytes after GM-CSF and G-CSF in septic neonates	On day 0, the HLA-DR expression of the septic neonates was significantly lower than the healthy control values (*p* < 0.0001, for both parameters). On follow-up (days 1, 3, and 5), a significant increase in HLA-DR expression was observed in all groups of septic neonates.
**IFN-γ**						
Payen et al. (2019) [85]	18 adults, 2 children	Multicenter case series	Three cohorts, collected in different periods	Both adults and children	The following were considered: monocyte expression of HLA-DR, lymphocyte immune-phenotyping, IL-6 and IL-10 plasma levels, bacterial cultures, disease severity, and mortality.	In 15 out of 18 patients, IFN-γ determined an increase in HLA-DR expression from 2666 [IQ 1547; 4991] to 12,451 [IQ 4166; 19,707], while the absolute number of lymphocyte subpopulations was not affected. Plasma levels of IL-6 (from 464 [201–770] to 108 [89–140] ng/mL (*p* = 0.04)) and IL-10 (from 29 [12–59] to 9 [1–15] pg/mL) decreased significantly. Three patients who received IFN-γ died. The other patients had clinical improvements (bacterial cultures became negative). The 2 pediatric cases improved rapidly, but 1 died due to hemorrhagic complications.
Tissières et al. (2012) [86]	70 neonates, 20 adults	Longitudinal study	Follow-up during the study	Both adults and neonates	Demonstrating that innate immune function is impaired in premature infants (particularly in ELBW). Assessing whether innate immune deficiency in extremely premature infants can be reversed by treatment with IFN-γ.	A 12 h course of ex vivo treatment of whole blood with IFN-γ restored the LPS responsiveness of circulating leukocytes in premature infants to levels measured in control adults (11.2 ± 4.5 ng/mL IL-6 in conditioned supernatants from IFN-γ-treated neonate leukocytes stimulated with LPS vs. 16.7 ± 2.8 in untreated leukocytes from healthy adults stimulated with LPS). In contrast, IL-10 cytokine level was decreased.
**GPX-4**						
Qu et al. (2023) [91]	283 (from four different datasets)	RCT	Follow-up during the study	Children	Assessing new biomarkers (involved in ferroptosis) in pediatric sepsis	GPX4 was markedly downregulated in sepsis in the training set relative to the control group (*p* < 0.05). The area under the curve (AUC) of the ROC of GPX4 in diagnosing sepsis was 0.64, with sensitivity and specificity of 0.79 and 0.5, respectively.

**Table 5:** A summary of some key points related to the knowledge gaps and research opportunities for immunomodulatory therapies in pediatric sepsis.

Subgroup	Biomarkers/Endpoints	Potential Interventions	Knowledge Gaps
Corticosteroids	Glucocorticoid receptor (GCR)	Corticosteroid therapy guided by endotype (A vs. B)	How to identify, at the bedside, children with sepsis who are likely to benefit from corticosteroid treatment, according to Wong’s endotype classification.
Sepsis with sHLH traits	Ferritin-soluble urokinase plasminogen receptor (suPAR)	Recombinant human IL-1 receptor antagonist (anakinra)	Role of anakinra in pediatric patients with sepsis and secondary hemophagocytic lymphohistiocytosis (sHLH) traits.
Pediatric septic shock	Organ dysfunction scores, mortality	IgM-enriched immunoglobulins	Can IgM-enriched immunoglobulins improve outcomes compared to standard IVIG in pediatric septic shock?
Sepsis with immune dysfunction	HLA-DR expression, leukocyte count	Immunostimulatory therapies (e.g., GM-CSF, IL-7), guided by PODIUM immune criteria	How to optimize immunostimulatory therapy in immune-dysregulated pediatric sepsis according to PODIUM-defined criteria.
Refractory septic shock	Organ dysfunction score, morbidity	Extracorporeal blood purification techniques	Can early extracorporeal purification reduce mortality, morbidity, and the need for ECMO in children with refractory septic shock?

## Abbreviations

AKI	Acute Kidney Injury
CI	Confidence Interval
CRRT	Continuous Renal Replacement Therapy
DIC	Disseminated Intravascular Coagulation
ELBW	Extremely Low Birth Weight
EOS	Early-Onset Sepsis
GCR	Glucocorticoid Receptor
G-CSF	Granulocyte Colony-Stimulating Factor
GLUT-1	Glucose Transporter 1
GM-CSF	Granulocyte-Macrophage Colony-Stimulating Factor
GPX4	Glutathione Peroxidase 4
HBD	Hepatobiliary Disfunction
HF	Hemofiltration
HLA	Human Leukocyte Antigen
HLH	Hemophagocytic Lymphohistiocytosis
HR	Hazard Ratio
HVHF	High-Volume Hemofiltration
ICU	Intensive Care Unit
IFN	Interferon
IL	Interleukin
IQR	Interquartile Range
IVIG	Intravenous Immunoglobulins
JAK	Janus Kinase
kDa	Kilodalton
LOS	Late-Onset Sepsis, Length of Stay
LPS	Lipopolysaccharide
MAS	Macrophage Activation Syndrome
MODS	Multiple-Organ Dysfunction Syndrome
MV	Mechanical Ventilation
NETs	Neuthrophil Extracellular Traps
NO	Nitric Oxide
OR	Odds Ratio
PD-1	Programmed Cell Death Protein 1
PD-L1	Programmed Death Ligand 1
PE	Plasma Separation Techniques
PEI	Polyethyleneimine
PICU	Pediatric Intensive Care Unit
PMX	Polymyxin
PRISM	Pediatric Risk of Mortality
RCT	Randomized Controlled Trial
rhIL-1ra	Recombinant Human IL-1 Receptor Antagonists
RR	Relative Risk
RRTs	Renal Replacement Therapies
ST	Surface Treatment
TAMOF	Thrombocytopenia-Associated Multiple-Organ Failure
Th	T Helper
TLR	Toll-Like Receptor
TNF	Tumor Necrosis Factor
TPE	Plasma Exchange
TSS	Toxic Shock Syndrome
